# Regulatory T cell therapy in lung transplantation: bridging the gap from bench to bedside

**DOI:** 10.3389/fimmu.2025.1654561

**Published:** 2025-10-22

**Authors:** Qianwei Li, Guorui Li, Jinteng Feng, Guangjian Zhang

**Affiliations:** ^1^ Department of Thoracic Surgery, The First Affiliated Hospital of Xi’an Jiaotong University, Xi’an, China; ^2^ Key Laboratory of Enhanced Recovery After Surgery of Intergrated Chinese and Western Medicine, Administration of Traditional Chinese Medicine of Shaanxi Province, The First Affiliated Hospital of Xi’an Jiaotong University, Xi’an, China

**Keywords:** regulatory T cells, lung transplantation, chronic lung allograft dysfunction, bronchiolitis obliterans syndrome, cellular therapy, immune tolerance

## Abstract

Lung transplant recipients face significantly poorer outcomes compared to other solid organ transplants, with median survival rates substantially lower despite current immunosuppressive regimens. Regulatory T cell (Treg) therapy has emerged as a promising approach for immune modulation, though its successful application in lung transplantation requires understanding of the unique pulmonary immune environment. This review examines how Tregs mediate immune responses in lung allografts and their role in rejection and tolerance pathways. We evaluate emerging evidence from preclinical studies of Treg therapy in lung transplantation, complemented by clinical experience from kidney and liver transplant trials that demonstrate safety and potential for reducing conventional immunosuppression. The analysis addresses key considerations for clinical implementation, including therapeutic strategies, timing of administration, and integration with existing protocols. This framework aims to guide the development of Treg-based therapies specifically tailored for lung transplant recipients.

## 1 Introduction

Lung transplantation outcomes continue to lag behind those of other solid organ transplants, with a median survival of only 6.5 years compared to >10 years for heart, kidney, and liver transplants (1). Even in more favorable patient populations, such as young bilateral lung transplant recipients or those with cystic fibrosis, median survival remains limited at 7.9 years. The current 5-year survival rate following lung transplantation is 50-60% (2). A major contributor to poor long-term outcomes is the high incidence of acute rejection in lung transplant recipients, which is strongly associated with the development of chronic lung allograft dysfunction (CLAD). CLAD manifests clinically as either bronchiolitis obliterans syndrome (BOS) or restrictive allograft syndrome (RAS), with BOS being the more common presentation and the leading cause of late mortality in this population (3).

While lifelong immunosuppression is the current standard of care for preventing rejection, it often comes at the cost of significant complications, including increased infection risk, hepatorenal toxicity, gastrointestinal side effects, myelosuppression, and malignancy (4). As a novel cellular immunotherapy, regulatory T cell (Treg) infusion has emerged as a promising approach for post-lung transplant immunomodulation, offering unique advantages over traditional immunosuppressive regimens.

In this review, we systematically examine the mechanisms and physiologic basis of Tregs in lung transplant immunity, critically evaluate current clinical translation efforts, and analyze existing strategies for Treg selection. Through this comprehensive synthesis, we highlight key knowledge gaps and discuss how current understanding may inform future therapeutic development for improving post-transplant outcomes.

## 2 Mechanisms of Treg-mediated immunosuppression and transplantation tolerance

Tregs are crucial for maintaining immune tolerance and preventing autoimmune pathologies. Initially characterized as CD25^+^ (IL-2 receptor α chain) CD4^+^ T cells capable of suppressing self-reactive and allogeneic responses, Tregs were later defined by the expression of FoxP3, a key transcriptional regulator essential for their development and function (5). Tregs can be classified into two main subgroups: natural Tregs (nTregs), which develop in the thymus and stably express FoxP3, and induced Tregs (iTregs), which differentiate from naive CD4^+^ T cells in peripheral tissues under the influence of cytokines like TGF-β and IL-2 (6).

**Figure 1:**
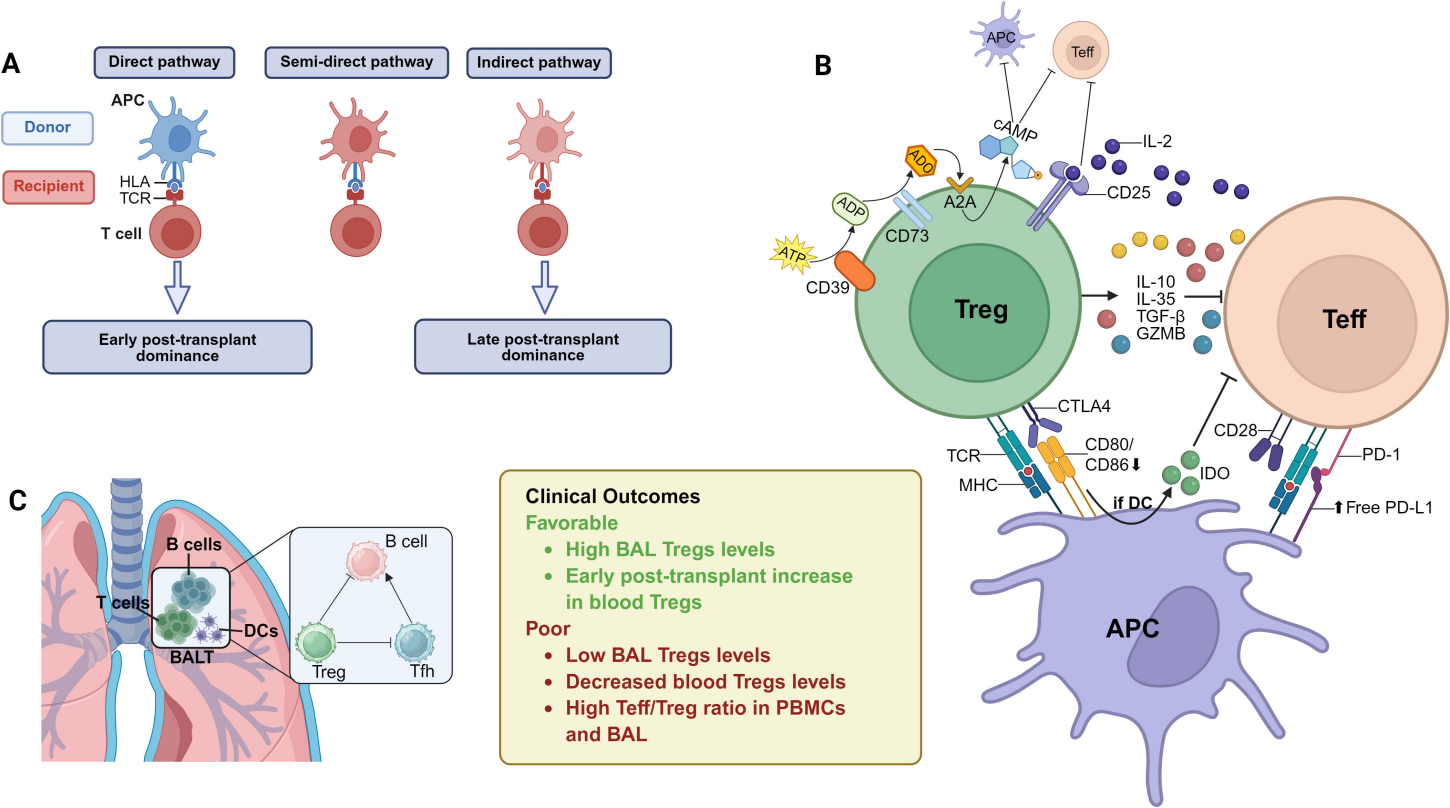
Regulatory T cell (Treg) dynamics and mechanisms in lung transplant outcomes. **(A)** Alloantigen recognition Pathways in lung transplantation: (1) Direct pathway: Donor APCs present HLA class I and II molecules to recipient CD8 and CD4 T cells; (2) Semi-direct pathway: Recipient APCs acquire donor MHC–peptide complexes and present them to recipient T cells; (3) Indirect pathway: Recipient APCs present donor-derived peptides via self HLA class I and II molecules to CD8 and CD4 T cells. **(B)** Treg-mediated immunosuppressive mechanisms: (1) Signal modulation: CTLA-4 downregulates CD80/CD86 on APCs and liberates PD-L1, which engages PD-1 on Teffs to reinforce inhibitory signaling. (2) Cytokine and cytolytic control: IL-10, TGF-β, and IL-35 reshape the microenvironment; CD25-mediated IL-2 consumption and GZMB-mediated cytolysis suppress Teff survival. (3) Metabolic regulation: CD39/CD73 produce ADO that suppresses APCs and Teffs, while DC-derived IDO depletes tryptophan to inhibit Teffs. **(C)** Tregs and clinical trajectories in lung transplantation: (1) FoxP3^+^ Tregs residing in BALT suppress both Tfh and B cells, preventing DSA production and promoting transplant tolerance. (2) Clinical correlations between Tregs and lung transplantation.

The pathological essence of allograft rejection lies in the host immune system’s activation of specific responses against donor antigens through three alloantigen recognition pathways: direct, indirect, and semi-direct (**Figure 1A**). Acute rejection is predominantly driven by direct recognition. Intact allogeneic HLA class I and II molecules expressed on donor antigen-presenting cells (APCs) directly interact with TCRs on recipient CD8 and CD4 T cells, respectively (7, 8). This mechanism predominates during the early post-transplant period when substantial numbers of donor APCs remain within the graft. Conventional models posit that donor APCs migration to secondary lymphoid organs constitutes a prerequisite for direct allorecognition. However, studies in mouse lung transplantation models have revealed a unique localized activation paradigm where donor CD11c^+^ dendritic cells (DCs) activate naive recipient T cells in the transplanted lung, forming early immunological synapses (9). Simultaneously, host APCs further activate T cells through indirect recognition (processing donor antigen peptide-self MHC complexes) and semi-direct recognition (capturing donor MHC-peptide complexes), collectively driving the acute rejection process (10). In the indirect pathway, donor-derived antigenic peptides are presented by recipient HLA class I and II molecules, thereby eliciting both CD4 and CD8 T-cell responses (11). Immunosuppressive medications routinely used after solid organ transplantation precisely exert their effects at different stages of allogeneic T cell activation (12). In contrast, chronic rejection is primarily mediated by the indirect pathway (13), occurring later post-transplantation and driving CLAD. This process results from persistent recipient immune cell infiltration into the graft or donor antigen retention in lymphoid tissues (14).

Tregs orchestrate transplantation tolerance through sophisticated multi-tiered mechanisms, with their primary function centered on active intervention in antigen presentation processes (**Figure 1B**). Tregs form immunological synapses with APCs, directly inhibiting APCs’ capacity to activate effector T cells (Teffs) and, upon disengaging, continue to exert immunosuppressive effects by removing antigen-MHC complexes from APCs’ surfaces, establishing persistent immunosuppression (15). Tregs exhibit high expression of cytotoxic T-lymphocyte-associated protein 4 (CTLA-4), which downregulate CD80/CD86 co-stimulatory molecules on APCs’ surfaces, not only attenuating the second signal required for Teffs activation but also triggering programmed death-ligand 1 (PD-L1) dissociation from CD80; the liberated PD-L1 subsequently binds to PD-1 receptors on Teffs’ surfaces, amplifying inhibitory signaling cascades (16). Furthermore, Tregs competitively deplete interleukin-2 (IL-2) through their high-affinity IL-2 receptor CD25, while simultaneously secreting immunosuppressive cytokines such as IL-10, transforming growth factor-beta (TGF-β), and IL-35, effectively remodeling the microenvironment and directly inducing Teffs apoptosis via granzyme/perforin pathways (17).From a metabolic perspective, Tregs express CD39 and CD73 on their surface, which collaboratively degrade extracellular adenosine triphosphate (ATP) to adenosine (ADO), resulting in increased ADO concentration within the microenvironment that inhibit both antigen presentation and Teff proliferation (18). 2,3-dioxygenase (IDO), which is produced by DCs upon Tregs’ CTLA-4 engagement of CD80 on DCs, contributes to the suppression of allogeneic T cell responses by depleting tryptophan (19).

## 3 Tregs in post-lung transplant immune responses

The dynamic changes in Tregs after lung transplantation are closely associated with transplant prognosis (**Figure 1C**). Studies in lung transplant patients have demonstrated that higher frequencies of Tregs in bronchoalveolar lavage fluid (BAL) within the first post-transplant year correlate inversely with acute cellular rejection (ACR) severity (20). Moreover, a low percentage (<3.2%) of CD4^+^FoxP3^+^ cells in BAL is a predictive factor for the development of BOS (21). In patients with CLAD, peripheral blood CD4^+^CD25^high^CD127^-^ Tregs are significantly reduced, with the degree of reduction related to the severity of BOS (22). The Teff/Treg ratio progressively increases from healthy controls to CLAD-free recipients and further to CLAD patients in both peripheral blood mononuclear cells (PBMCs) and BAL (23), highlighting the importance of Teff activation and Treg functional suppression in post-transplant immune responses. Notably, an increased proportion of Tregs (CD4^+^CD25^high^) expressing CD127^low^, FoxP3^+^, and IL-2^+^ in peripheral blood at 3 weeks post-transplantation has an independent protective effect against the development of CLAD within 2 years (24).

Discrepancies persist regarding Treg quantity versus functional relevance. In asymptomatic lung transplant recipients experiencing their first A1-grade rejection episode, no significant differences were found in the Treg content or the degree of FoxP3 gene demethylation in lung tissue between patients who developed CLAD within 2 years and those who remained CLAD-free for over 5 years. Instead, an increased CD8^+^ T cell infiltration may indicate a poorer prognosis (25). This discrepancy underscores that Tregs’ functional competence – exemplified by CTLA-4-mediated CD80/CD86 internalization, CD39/CD73-dependent ADO production, or IDO-driven tryptophan depletion by DCs upon CTLA-4 engagement of CD80 – rather than numerical prevalence, likely dictates clinical outcomes. Critical knowledge gaps persist regarding the longitudinal evolution of Tregs within the allograft microenvironment, necessitating integrated single-cell transcriptomic profiling coupled with functional validation to elucidate their long-term functional dynamics and therapeutic modulation potential.

In lung transplantation, Tregs function in a unique tissue-resident-dependent manner, and their immune regulation is closely linked to microenvironmental remodeling of bronchus-associated lymphoid tissue (BALT) (**Figure 1C**). Successfully tolerized lung allografts contain abundant FoxP3^+^ T cells within BALT. Patients whose lung allografts harbor lymphoid aggregates rich in FoxP3^+^ T cells remain rejection-free for at least 6 months post-transplantation (26). PD-L1 signaling, as demonstrated in murine studies, is essential for the induction and stability of FoxP3^+^ Tregs, providing a mechanistic basis for sustained local immune tolerance in lung allografts (27). Conversely, selective depletion of graft-resident FoxP3^+^ T lymphocytes in mouse lung transplantation models disrupts immune tolerance and upregulates CXCL13 expression in the graft interstitium. CXCL13 binds to CXCR5, mediating recruitment and persistent interactions between CXCR5^+^ follicular helper T cells (Tfhs) and B cells, which drives donor-specific antibody (DSA) production and ultimately antibody-mediated rejection (AMR) (26, 28).

Although ACR after lung transplantation can initiate in the absence of recipient secondary lymphoid organs, murine studies indicate that progression from ACR to RAS-like fibrosis requires the spleen and other secondary lymphoid structures (29), highlighting a dual-compartment model in which early events are lung-specific, while the progression to fibrosis depends on peripheral immune priming. Among the intrinsic factors of the lung, the resident microbiota colonizing the organ imparts a higher baseline immunogenicity to the transplanted lung compared to relatively “sterile” organs such as the heart or kidney. Post-transplant, the lung microbiota is frequently dysbiotic and continuously reseeded from the upper respiratory tract, which is associated with inflammation, fibrosis, and increased chronic rejection risk (30). In murine models, different microbiota significantly influence CD4^+^FoxP3^+^ Treg levels, thereby determining the severity of transplant rejection; antibiotic disruption of the microbiota reduces Tregs and exacerbates rejection (31). Although no direct clinical studies currently link lung microbiota, Treg-mediated tolerance, and lung transplant outcomes, existing evidence suggests that dysbiosis may impair Treg function (32), thereby increasing the risk of CLAD and rejection (33, 34). Therefore, future studies should explore how the microbiota modulates Treg function and influences immune tolerance and rejection following lung transplantation.

## 4 Therapeutic applications of Tregs in lung transplantation

### 4.1 Preclinical model advancements

To date, only a few animal studies have reported on the application of Tregs in lung transplantation. IL-2/anti-IL-2 monoclonal antibody complexes (IL-2cx), which preferentially activate IL-2Rα signaling, robustly expand Tregs in both lymphoid and non-lymphoid organs. Short-term, high-dose IL-2cx preconditioning induces the formation of inducible tertiary lymphoid structures (iTLS) within lung allografts. Within two weeks post-transplantation, FoxP3^+^ Tregs predominantly proliferate and are activated within iTLS, suppressing Th1/Th17 polarization to establish immune tolerance (35).

In another study, ex vivo-expanded Tregs were infused into rat lung transplant models (a fully MHC-mismatched F344→WKy rat orthotopic left lung transplant model) or human lungs deemed unsuitable for transplantation due to edema or poor compliance during ex vivo lung perfusion (EVLP). Results demonstrated that infused Tregs effectively infiltrated lung parenchyma while retaining suppressive capacity, suggesting their potential for pre-transplant local immunomodulation. However, Treg retention within grafts lasted only 3 days, with no significant difference in acute rejection rates between Treg-treated and control groups by day 7, suggesting that sustained Treg residency is critical for tolerance. While overexpression of homing receptors like CCR4 may improve graft homing, the primary issue is the persistence of transferred Tregs (36), as they often die *in vivo* due to IL-2 deprivation after ex vivo culture, despite their survival during expansion *in vitro (* 37).

In a recent mouse lung retransplantation model, it was shown that graft-resident FoxP3^+^ cells are maintained by continuous recruitment from the thymus. Blocking the recruitment of these FoxP3^+^ cells led to both AMR and ACR. Moreover, local administration of IL-33 has been demonstrated to effectively expand and activate these graft-resident FoxP3^+^ cells, potentially resolving the issues of sustained Treg residency and survival in the graft (38).

Despite these insights, the mechanisms governing Treg migration, persistence, and engraftment in lung allografts remain elusive. Future studies should integrate intravital imaging and single-cell sequencing to further elucidate Treg dynamics and their survival mechanisms within the graft.

### 4.2 Clinical progress: lessons from kidney and liver transplantation

While Treg-based therapy has not yet been tested in clinical trials of lung transplantation, advances in kidney and liver transplantation (summarized in **Table 1**) provide critical benchmarks, demonstrating reduced rejection rates and stable graft function. However, caution is required when extrapolating these findings to lung transplantation, owing to its distinct immune microenvironment. Nevertheless, these cross-disciplinary insights provide a framework for designing Treg-based trials in lung transplantation, as outlined in the following sections.

**Table 1:** Published studies on Tregs infusion in solid organ transplantation.

Trial id	Phase	Clinical setting	Product, dose and infusion timing	Combined immunosuppressive regimen	Outcomes and safety
NCT02088931 (39)	I	kidney transplantation (n=3) 1-year follow-up	• Polyclonal CD4^+^CD25^+^CD127^low/−^ nTreg • 320 × 10^6^ cells/recipient • 6 months post-transplant	Induction: Basiliximab Tacrolimus (TAC), mycophenolate (MMF), and prednisone (PRED)	Well-tolerated infusion in all patients 100% patient and graft survival at 1 year 1 case of self-limiting neutropenia Graft inflammation improved in 2/3 patients 1 patient with persistent inflammation (pre-existing DSA before the infusion)
NCT02371434 (40)	I/IIa	Kidney transplantation (n=11) 60-week follow-up Control group (n=9): Basiliximab + TAC/MMF/PSL	• Polyclonal CD4^+^CD25^+^ FoxP3^+^ nTreg • 0.5, 1.0, or 2.5-3.0×10^6^ cells/kg body weight • 7 days after transplantation	Absence of basiliximab induction Prednisolone (PSL): 500 mg (Day 0) → 125 mg/day (Day 1) → 20 mg/day (Days 2-14) → tapered to 2.5 mg/day (Weeks 13-14), then ceased MMF: 2 g/day (Days 1-14) → 1 g/day (Weeks 3-36) → tapered from Week 37, ceased by Week 48 TAC (target trough): 12 ng/mL (Weeks 0-2) → 10 ng/mL (Weeks 3-12) → 8 ng/mL (Weeks 13-36) → 6 ng/mL (Week 37 onward) Weeks 49+: TAC monotherapy	Well-tolerated cell infusion in all recipients All patients: Stable graft function 8/11 (72.7%): Low-dose tacrolimus monotherapy (<6 ng/mL) within 48 weeks 3/11 (27.3%): Returned to triple therapy due to acute rejection Control group: 4/9 (44.4%): Dual immunosuppressive therapy 5/9 (55.6%): Triple immunosuppressive therapy
NCT02129881 (41)	I	Kidney transplantation (n=12) 4-year follow-up Control group (n=19): Basiliximab + TAC/MMF/PSL	• Polyclonal nTreg • 1-10×10^6^ cells/kg body weight • 5 days after transplantation	Absence of basiliximab induction PSL: 500 mg (Day 0) → 125 mg/day (Day 1) → 20 mg/day (Days 2-14) → tapered to 2.5 mg/day (Weeks 13-14), then ceased MMF: 0.5 g/day (Days -7 to -2 pre-transplant) → 2 g/day (Day -1 to Day 14) → 1 g/day (Weeks 3-36) → tapered from Week 37, ceased by Week 48 TAC (target trough): 3–12 ng/mL (Days -2 to 14) → 3–10 ng/mL (Weeks 3-12) → 3–8 ng/mL (Weeks 13-36) → 3–6 ng/mL (Week 37 onward) Week 49+: TAC monotherapy	Well-tolerated in all recipients 100% rejection-free graft survival at 48 months vs 78.9% in control 4 patients discontinued mycophenolate, transitioned to tacrolimus monotherapy
NCT02145325 (42)	I	Kidney transplantation (n=9) 2-year follow-up Control group: Alemtuzumab + SRL/TAC/MMF/CS	• Polyclonal CD4^+^CD25^+^CD127^−^FOXP3^+^ nTreg • 0.5, 1.0, or 5.0 × 10^9^ cells/recipent • 60 days after transplantation	Induction: Alemtuzumab Corticosteroids (CS): 500 mg (Day 0) → 250 mg/day (Day 1) → 125 mg/day (Day 2), then ceased MMF: 1.44-1.8 g/day (Starting Day -2) TAC (target trough): 8–12 ng/mL (Days -2 to 29) Sirolimus (SRL) (target trough): 8–12 ng/mL (Starting Day 30)	Well-tolerated in all recipients 100% patient/graft survival over 2 years No rejection or DSA at 3 months At 1 year, 1 subclinical rejection due to poor adherence, 1 DSA case with leukopenia and disease recurrence
NCT02091232 NCT01656135 (43)	I/II	Kidney transplantation (n=3) Follow-up: >6 years Control group (n=3): Basiliximab + TAC/MMF/PSL	• Monoclonal CD4^+^CD25^+^CD127^low^ darTreg • 0.86, 1.1 or 1.9 × 10^4^ cells/kg body weight • 7–11 days after transplantation	Absence of basiliximab induction PSL: Discontinued within 14 weeks post-transplant MMF: Discontinued at 11–13 months post-transplant, following a rejection-free protocol biopsy at 8 months and stable renal function TAC: 3–12 ng/mL (Days -4) → 3–6 ng/mL (Months 9). Complete immunosuppression withdrawal not permitted	Well-tolerated in all recipients No rejection on protocol biopsies >6 years posttransplant: all on tacrolimus monotherapy, excellent graft function, no rejection episodes
UMIN-000015789 (44)	I/IIa	Liver transplantation (n=10) Follow-up: up to 51 months (longest)	• Monoclonal CD4^+^CD25^+^ FOXP3^+^ iTreg • 3.39 ± 2.12 × 10^6^ cells/kg body weight • 13 days after transplantation	Cyclophosphamide (CTX): 40mg/kg (Days 5) Methylprednisolone (mPSL): 20 mg/day (stopped within 1 month) MMF: 0.5-1.5g/day (stopped within 1 month). TAC: Weaning started at 6 months post-transplant, ceased by 18 months if graft function stable for 3 months	Well-tolerated in all recipients 7 patients successfully weaned off immunosuppression 3 patients with immunological liver diseases developed ACR during weaning, then stable on reduced immunosuppression
NCT02166177 (45)	I	Liver transplantation (n=9) Follow-up: 6 or 12 months	• CD4^+^FOXP3^+^ nTreg • 0.5–1 or 3-4.5 ×10^6^ cells/kg body weight • 3–16 months after transplantation	Induction: Thymoglobulin (ATG) mPSL: 500 mg (Day 0) → tapered and stopped by Weeks 10 TAC: 5–8 ng/mL (Starting Day 1) → 2–5 ng/mL (from Weeks 6-8) SRL: Started at Weeks 6-8 (trough 5–8 ng/mL) → Final levels: Tacrolimus 2–5 ng/mL, Sirolimus 2–8 ng/mL	Well-tolerated in all recipients One patient (4.5 × 10^6 Tregs/kg) experienced transient fever, temporary neutropenia, lymphopenia, and mild liver function impairment
NCT02474199 (46)	I/II	Liver transplantation (n=5) Follow-up: up to 48 months (longest)	• Monoclonal darTreg • 1–3 or 3-5 ×10^8^ cells/recipent • 2–6 years after transplantation	Calcineurin inhibitor: Dose reduced to 67% before darTreg infusion Post-infusion: Further reduction in calcineurin inhibitor dose	Well-tolerated in all recipients 2 out of 5 patients reduced calcineurin inhibitors by 75% and stopped second immunosuppressant 4 out of 5 patients experienced acute rejection

### 4.3 Treg product development and selection

#### 4.3.1 Polyclonal Tregs: standardization vs limitations

Polyclonal Tregs represent a cell population derived from the ex vivo expansion of nTregs (CD4^+^CD25^high^CD127^low^FoxP3^+^) isolated from peripheral blood. Standard expansion protocols utilize anti-CD3/CD28 antibody stimulation combined with IL-2, whereas rapamycin supplementation inhibits conventional T cell proliferation and maintains high FoxP3 expression during expansion (47).

Despite their well-established manufacturing protocols, which have made them the most widely used cellular product in clinical trials, polyclonal Tregs face significant translational challenges. Their non-antigen-specific nature compromises homing and retention efficiencies in grafts, limiting local immunomodulatory effects (48). Additionally, while rapamycin is essential for maintaining Treg purity, it reduces expansion efficiency, necessitating repeated anti-CD3/CD28 stimulation and extended culture periods. This prolonged cultivation can lead to diminished FoxP3 expression, even in high-purity Tregs, after multiple rounds of stimulation (49). Nevertheless, safety assessments and preliminary efficacy data from polyclonal Treg therapies have laid a crucial foundation for developing optimized cellular products.

#### 4.3.2 Donor antigen-specific Tregs (darTregs): precision challenges

DarTreg preparation involves ex vivo stimulation and expansion of recipient-derived Tregs using donor APCs, including DCs, B cells, or unfractionated PBMCs. *In vitro* studies demonstrate that darTregs exert significantly stronger suppression of alloantigen-driven proliferation, exhibiting 5- to 32-fold enhanced potency relative to polyclonal Tregs (50).

Nevertheless, multiple technical barriers impede the clinical translation of darTreg therapies. The foremost limitation is the donor APC requirement, posing significant challenges for deceased-donor lung transplantation. Moreover, existing stimulation protocols demonstrate suboptimal efficacy, with only ≤10% of alloantigen-specific Tregs undergoing successful activation and expansion (51). The absence of standardized isolation and expansion protocols further exacerbates these limitations, substantially impeding clinical adoption of darTreg-based therapies.

#### 4.3.3 Chimeric antigen receptor regulatory T cells (CAR-Tregs): engineered solutions

CAR-Treg infusion therapy represents a novel approach to enhance Treg enrichment in transplanted organs, addressing both the insufficient specificity of polyclonal Tregs and the low expansion efficiency of darTregs, while considering the unique physiology of lung allografts.

These genetically engineered Tregs carry a chimeric antigen receptor (CAR) on their surface, enabling specific target antigen recognition. The CAR structure comprises an extracellular antigen-binding domain (typically derived from an antibody’s single-chain variable fragment), a transmembrane domain, and an intracellular signaling domain (52). In transplantation settings, the human leukocyte antigen HLA-A2 serves as a common CAR target. As donor-recipient mismatched MHC molecules are exclusively expressed in the graft, CAR-Tregs can specifically recognize donor HLA and accumulate in the transplanted organ (53).

Preclinical studies have demonstrated the superior capability of CAR-Tregs in graft homing and retention compared with polyclonal Tregs, along with more effective suppression of alloimmune-mediated injury (54). Notably, in heterotopic heart transplantation models with either single HLA-A2 mismatch or multiple MHC mismatches, adoptive transfer of HLA-A2-specific CAR Tregs significantly prolonged graft survival (55). These findings provide strong preclinical support for CAR-Treg application in lung transplantation.

Clinical development of CAR-Tregs in transplantation remains at an early stage. Two ongoing phase I/II multicenter open-label clinical trials (NCT04817774 and NCT05234190) are assessing the safety and efficacy of HLA-A2-specific CAR-Tregs in HLA-A2-mismatched kidney and liver transplantation, respectively. While NCT04817774 has completed enrollment and remains active, data are still pending. NCT05234190 continues to recruit participants.

Although preclinical studies support the therapeutic potential of CAR-Tregs across various transplantation models, further experimental evidence specific to lung transplantation is required. Prior to clinical translation, a systematic assessment of the homing capacity and immunomodulatory functions of CAR-Tregs in lung allograft models - using both single HLA-A2 mismatch and multiple MHC mismatch systems - would provide more robust preclinical evidence.

### 4.4 Clinical trial framework for lung transplantation

#### 4.4.1 Spatiotemporally precise intervention

Strategic timing of Treg administration is critical for therapeutic outcomes. For ACR, peaking at 2–4 weeks post-transplantation (56), prophylactic Treg administration (2–3×10^6^ cells/kg) prior to this high-risk window to suppress effector T cell activation.

For AMR typically developing within the first year post-transplant (57), therapeutic intervention is optimal in patients with AMR occurring at 6–12 months, as this timing: (i) avoids interference with induction therapies (e.g., anti-IL-2R antibodies); (ii) minimizes early calcineurin inhibitor (CNI) exposure (2); and (iii) coincides with AMR’s established association with CLAD progression and treatment refractoriness (58).

#### 4.4.2 Innovative monitoring system

The unique immunological landscape of lung transplantation—characterized by (i) heightened rejection risk in single-lung grafts, (ii) predominance of donor-derived DCs, and (iii) persistent environmental antigen exposure of the allograft (59)—mandates establishment of a comprehensive monitoring framework for evaluating Treg therapy efficacy.

Standardized monitoring should incorporate serial pulmonary function tests, radiographic imaging, and surveillance bronchoscopy with transbronchial biopsies. Primary endpoints must evaluate treatment safety and feasibility, whereas secondary endpoints should include: (a) biopsy-proven rejection rates, (b) extent of immunosuppression reduction, and (c) longitudinal changes in tolerance-associated biomarkers. Cutting-edge methodologies combine single-cell RNA sequencing (scRNA-seq) for clonal tracking of administered Tregs with spatial transcriptomics to map functional subsets within BALT.

#### 4.4.3 Immunosuppression optimization

Immunosuppression optimization is critical for Treg infusion trials. Current clinical protocols typically combine induction therapy (including basiliximab, ATG, or alemtuzumab) with triple maintenance immunosuppression consisting of: (i) CNI (TAC or CTX), (ii) MMF, and (iii) corticosteroids (60). Notably, more than 80% of adult lung transplant recipients receive induction therapy, with IL-2R antagonists (primarily basiliximab) accounting for over 70% of cases (61).

Current evidence suggests avoiding basiliximab in Treg trials, as its IL-2 receptor blockade may impair Treg function (62). Alternative induction strategies, such as alemtuzumab, ATG, or induction-free protocols, have shown better compatibility with adoptive Treg transfer (63). Furthermore, a gradual transition from CNI to mTOR inhibitors (e.g., sirolimus) may improve Treg survival and functionality (42). The successful incorporation of these modifications into Treg-based therapeutic protocols represents a key challenge in clinical trial design.

## 5 Conclusion

Lung transplant recipients face significantly inferior long-term survival compared to other solid organ transplants, necessitating a paradigm shift beyond conventional immunosuppression. This review systematically demonstrates the potential of regulatory T cell therapy to induce transplant tolerance through multi-layered immunomodulatory mechanisms, representing a transformative evolution from broad-spectrum immunosuppression to targeted immune remodeling in pulmonary transplantation. While Treg therapy has shown feasibility in reducing reliance on traditional immunosuppressants in kidney and liver transplantation, its application in lung transplantation confronts unique challenges, including: (i) the lung’s distinctive immunologic profile driven by continuous environmental antigen exposure; (ii) the dynamic development of tertiary lymphoid structures within the allograft; and (iii) limited therapeutic efficacy due to the suboptimal homing efficiency and restricted persistence of current Treg products.

Clinical translation should adopt a stepwise approach, progressing from safety validation of polyclonal Tregs to genetically engineered antigen-specific Treg formulations, combined with the development of lung-selective delivery systems and noninvasive biomarker monitoring platforms. Future investigations should prioritize: (a) elucidation of spatiotemporal dynamics among pulmonary Treg subsets; (b) optimization of immunosuppressive regimen compatibility; and (c) clarification of how the lung microbiota shapes Treg function and thereby influences rejection or tolerance. These advancements promise to catalyze a strategic transition from passive immunosuppression to active immune tolerance induction, potentially revolutionizing long-term outcomes in lung transplantation.

## Glossary

Treg: Regulatory T cell
CLAD: Chronic lung allograft dysfunction
BOS: Bronchiolitis obliterans syndrome
RAS: Restrictive allograft syndrome
nTregs: Natural regulatory T cells
iTregs: Induced regulatory T cells
FoxP3: Forkheadbox protein 3
TGF-β: Transforming growth factor-β
IL-2: Interleukin-2
APCs: Antigen-presenting cells
DCs: Dendritic cells
Teffs: Effector T cells
PD-L1: Programmed death-ligand 1
ATP: Triphosphate
ADO: Adenosine
IDO: 2,3-dioxygenase
CTLA-4: Cytotoxic T lymphocyte antigen-4
TIGIT: T cell immunoreceptor with immunoglobulin and ITIM domains
BAL: Bronchoalveolar lavage fluid
ACR: Acute cellular rejection
PBMCs: Peripheral blood mononuclear cells
MHC: Major histocompatibility complex
BALT: Bronchus-associated lymphoid tissue
Tfhs: Follicular helper T cells
DSA: Donor-specific antibodies
AMR: antibody-mediated rejection
GZMB: Granzyme B
IL-2cx: IL-2/anti-IL-2 mAb complexes
iTLS: Inducible tertiary lymphoid structures
EVLP: Ex vivo lung perfusion
ATG: Anti-thymocyte globulin
darTregs: Donor antigen-specific regulatory T cells
CAR-Tregs: Chimeric antigen receptor regulatory T cells
TAC: Tacrolimus
MMF: Mycophenolate
PRED: Prednisone
PSL: Prednisolone
mPSL: Methylprednisolone
CTX: Cyclophosphamide
SRL: Sirolimus
CS: Corticosteroids
ATG: Thymoglobulin
CNI: Calcineurin inhibitor
scRNA-seq: Single-cell RNA sequencing
